# Crystal structure of DNA polymerase I from *Thermus* phage G20c

**DOI:** 10.1107/S2059798322009895

**Published:** 2022-10-27

**Authors:** Josefin Ahlqvist, Javier A. Linares-Pastén, Andrius Jasilionis, Martin Welin, Maria Håkansson, L. Anders Svensson, Lei Wang, Hildegard Watzlawick, Arnþór Ævarsson, Ólafur H. Friðjónsson, Guðmundur Ó. Hreggviðsson, Bernd Ketelsen Striberny, Eirin Glomsaker, Olav Lanes, Salam Al-Karadaghi, Eva Nordberg Karlsson

**Affiliations:** a Division of Biotechnology, Department of Chemistry, Lund University, PO Box 124, 221 00 Lund, Sweden; b SARomics Biostructures (Sweden), Medicon Village, 223 81 Lund, Sweden; c Institute of Biomedical Genetics, University of Stuttgart, Allmandring 31, 70569 Stuttgart, Germany; d Matís, Vínlandsleið 12, 113 Reykjavík, Iceland; e Department of Biology, School of Engineering and Natural Sciences, University of Iceland, Sturlugata 7, 102 Reykjavík, Iceland; f ArcticZymes Technologies, PO Box 6463, 9294 Tromsø, Norway; University of Western Australia, Crawley, Australia

**Keywords:** DNA polymerase I, *Thermus* phage G20c, *Thermus thermophilus*, thermophilic bacteriophages, structural motifs, PolI_G20c

## Abstract

This study describes the structure of DNA polymerase I from *Thermus* phage G20c, termed PolI_G20c. This is the first structure of a DNA polymerase originating from a group of related thermophilic bacteriophages infecting *Thermus thermophilus*, including phages G20c, TSP4, P74-26, P23-45 and phiFA and the novel phage Tth15-6. Sequence and structural analysis of PolI_G20c revealed a 3′–5′ exonuclease domain and a DNA polymerase domain, and activity screening confirmed that both domains were functional. No functional 5′–3′ exonuclease domain was present. Structural analysis also revealed a novel specific structure motif, here termed SβαR, that was not previously identified in any polymerase belonging to the DNA polymerases I (or the DNA polymerase A family). The SβαR motif did not show any homology to the sequences or structures of known DNA polymerases. The exception was the sequence conservation of the residues in this motif in putative DNA polymerases encoded in the genomes of a group of thermophilic phages related to *Thermus* phage G20c. The structure of PolI_G20c was determined with the aid of another structure that was determined in parallel and was used as a model for molecular replacement. This other structure was of a 3′–5′ exonuclease termed ExnV1. The cloned and expressed gene encoding ExnV1 was isolated from a thermophilic virus metagenome that was collected from several hot springs in Iceland. The structure of ExnV1, which contains the novel SβαR motif, was first determined to 2.19 Å resolution. With these data at hand, the structure of PolI_G20c was determined to 2.97 Å resolution. The structures of PolI_G20c and ExnV1 are most similar to those of the Klenow fragment of DNA polymerase I (PDB entry 2kzz) from *Escherichia coli*, DNA polymerase I from *Geobacillus stearo­thermophilus* (PDB entry 1knc) and *Taq* polymerase (PDB entry 1bgx) from *Thermus aquaticus*.

## Abstract 2

The crystal structure of DNA polymerase I from *Thermus* phage G20c is presented and represents the first crystal structure of DNA polymerase I from a group of related thermophilic bacteriophages. The structure reveals a new structural motif termed SβαR that was not previously identified in other DNA polymerases I (or in the DNA polymerase A family).

## 1. Introduction

Arthur Kornberg and coworkers described the first polymerase in 1956 (Kornberg, Kornberg *et al.*, 1956 ▸; Kornberg, Lehman *et al.*, 1956 ▸). This DNA polymerase was later classified as a DNA polymerase I, which is one of the enzymes that participates in DNA replication of prokaryotes (Worthington & Worthington, 2011 ▸). There are several types of complementary polymerases that act on DNA and/or RNA, and combined they ensure all DNA and RNA replication and repair processes required for each species. The number of DNA polymerases in a given organism can be correlated to the complexity of its genome and replication process. Thus, viruses generally only have one DNA polymerase, while prokaryotic cells generally possess five different DNA polymerases and eukaryotic cells can contain up to 15 different DNA polymerases (Maga, 2019 ▸). Based on sequence similarity, the polymerases are separated into seven families (A, B, C, D, X, Y and RT), and polymerases of the same family may appear in several species (Ishino & Ishino, 2014 ▸). DNA polymerase I, which belongs to polymerase family A (Delarue *et al.*, 1990 ▸), is one of the most abundant polymerase types in prokaryotes. One of its main functions is to fill the gaps in DNA that arise during DNA replication, repair and recombination according to the general DNA polymerase reaction dNTP + DNA_*n*_ ⇌ PP_i_ + DNA_*n*+1_, as described previously (Kornberg, Kornberg *et al.*, 1956 ▸; Kornberg, Lehman *et al.*, 1956 ▸; Worthington & Worthington, 2011 ▸; Steitz, 1998 ▸; Choi, 2012 ▸).

The active site of DNA polymerase I has binding sites for a template strand, the DNA primer terminus (the initiation ‘i’ site) and the incoming dNTP (the ‘i + 1’ site). The third residue in the Asp–Glu–Asp catalytic triad coordinates two divalent metal ions (either Mg^2+^ or Mn^2+^) that stabilize the charge and geometry during the nucleotidyl-transfer reaction. One of the ions binds the 3′-hydroxyl (3′-OH) of the terminal DNA primer at the ‘i’ site and decreases the p*K*_a_ of the 3′-OH, facilitating nucleophilic attack of 3′-O on the α-phosphate of the incoming nucleotide. The other metal ion binds the phosphates of the incoming dNTP at the ‘i + 1’ site, positioning the incoming dNTP and stabilizing the PP_i_ leaving group. The 3′-OH of the DNA primer terminus then attacks the α-phosphate of the dNTP and a new phosphodiester bond is formed with release of PP_i_, after which the newly formed DNA primer terminus translocates by one base from the ‘i + 1’ site to the ‘i’ site and the nucleotidyl-transfer reaction is repeated (Steitz, 1998 ▸; Choi, 2012 ▸).

Moreover, DNA polymerase I enzymes have 3′–5′ exo­nuclease activity for proofreading that excises mis-incorporated nucleotides to ensure that an accurate sequence is synthesized (Hashimoto *et al.*, 2001 ▸), as well as 5′–3′ exo­nuclease activity that enables the removal of deoxy­ribonucleotides and ribonucleotide primers (on the lagging strand) during DNA replication (Bhagavan & Ha, 2015 ▸). This latter activity has also been described as 5′ nuclease (Lyamichev *et al.*, 1993 ▸) or more commonly as flap endonuclease or FEN activity (Harrington & Lieber, 1994 ▸; Xie & Sayers, 2011 ▸).

In general, the known DNA polymerases in this group have a highly conserved structure, which usually indicates important, irreplaceable functions in the cell, conserving them in evolution. Their overall catalytic subunits vary very little from species to species and their structural organization can be described as resembling a right hand, with thumb, fingers and palm domains (Perler *et al.*, 1996 ▸; Kohlstaedt *et al.*, 1992 ▸). The palm domain in particular is extremely conserved in most families, while the fingers and thumb domains are more variable (Maga, 2019 ▸). The DNA is bound to the palm domain when the enzyme is active, and appears to catalyse transfer of the phosphoryl group. The function of the finger domain is to bind dNTPs, while the thumb domain plays a potential role in the processivity, translocation and positioning of the DNA (Steitz, 1999 ▸). The processivity refers to the average number of nucleotides that are added before the polymerase needs to release its DNA template. Replicative DNA polymerases have a processivity of the order of 10^2^–10^3^ nucleotides, which can be further increased up to 10^5^–10^6^ by the action of auxiliary proteins (Watson, 2004 ▸; Maga, 2019 ▸).

The studied DNA polymerase, PolI_G20c, originates from the thermophilic bacteriophage G20c that infects *Thermus thermophilus* (Xu *et al.*, 2017 ▸). *T. thermophilus* is a Gram-negative, thermophilic heterotrophic bacterium that is found in coastal hot springs all around the world. PolI_G20c and homologous enzymes originating from evolutionarily related thermophilic bacteriophages are all annotated as DNA polymerases I in the NCBI database. All of these polymerases have the typical domain organization of DNA polymerases I; however, our investigations show that they all lack the N-terminally located part of the 5′–3′ exonuclease domain. This feature is analogous to the Klenow fragment (KF), which is a cleaved part corresponding to a 605-amino-acid sequence starting at residue 324 in DNA polymerase I from *Escherichia coli* (UniProt accession P00582). Here, we present the first structure and activity study of the DNA polymerase from *Thermus* phage G20c, isolated from Geyser Valley, Kamchatka, Russia (Loredo-Varela *et al.*, 2013 ▸), which is homologous to the putative DNA polymerase I encoded in the genomes of several long-tailed bacteriophages, including the *Thermus* viruses P74-26 from Uzon Valley, Kamchatka, Russia and P23-45 from Geyser Valley, Kamchatka, Russia (Minakhin *et al.*, 2008 ▸), *Thermus* phage Tth15-6 from a coastal hot spring at Reykjanes in Isafjarddjup, Iceland (Ahlqvist *et al.*, 2022 ▸), *Thermus* phage TSP4 from hot springs in Tengchong, People’s Republic of China (Lin *et al.*, 2010 ▸) and *Thermus* phage phiFa from Mount Vesuvius, Italy (Lopatina *et al.*, 2019 ▸). The structure of PolI_G20c was solved with the aid of the structure of an exonuclease termed ExnV1, which was determined in parallel in this work and originates from an Icelandic virus metagenome collected from several hot springs in Iceland. The gene coding for ExnV1 may be part of a truncated gene that originally encoded a DNA polymerase I. A novel structural motif, SβαR, that was not previously associated with DNA polymerases I was identified in the solved structures of ExnV1 and PolI_G20c. Based on amino-acid sequence alignments, this motif is likely to be found in the structures of the DNA polymerases from the related bacterio­phages described above.

## 2. Materials and methods

### 2.1. Gene identification and cloning

The nucleotide sequence of *Thermus* phage G20c gene *G20c_11* (GenBank accession KX987127.1) encoding PolI_G20c (GenBank accession API81819.1; Xu *et al.*, 2017 ▸) was optimized for expression in *E. coli*. The synthetic gene sequence (GenBank accession ON338039; GenScript Biotech) was inserted between NdeI and XhoI restriction sites into the pET-21b(+) vector (Novagen), resulting in the construct pET21b::PolI_G20c. The sequence was cloned in frame with the C-terminal hexahistidine tag encoded by the expression vector.

The gene (GenBank accession OK094307) encoding ExnV1 was identified in an Icelandic virus metagenome by manually curated automated annotation of the sequence data set termed matis_pooled_all accumulated from sequence data sets from several sampling sites in terrestrial hot springs located in the south of Iceland, as described by Aevarsson *et al.* (2021 ▸). The codon usage was optimized for expression in *E. coli* and the synthetic gene sequence (GenBank accession ON489251; General Biosystems) was inserted between BamHI and BsrGI restriction sites into the rhamnose-inducible vector pJOE5751.1 (Wegerer *et al.*, 2008 ▸), resulting in the construct pLEI447.1. The gene sequence was cloned in frame with the N-terminal hexahistidine tag encoded by the expression vector.

### 2.2. Protein production

Recombinant PolI_G20c was produced in *E. coli* Origami (DE3) cells (Novagen) heat-shock transformed with the expression construct. The cells were grown in baffled Erlenmeyer flasks in Lysogeny Broth (Lennox) (LB) medium supplemented with 100 µg ml^−1^ ampicillin at 37°C. Heterologous overexpression of PolI_G20c was induced with 1 m*M* isopropyl β-d-1-thiogalactopyranoside when the culture reached an optical density at 600 nm (OD_600 nm_) of 0.3. Induction was performed for 4 h at 20°C and 200 rev min^−1^.

Recombinant ExnV1 was produced in *E. coli* JM109 cells (Promega) heat-shock transformed with the expression construct. The cells were grown in baffled Erlenmeyer flasks in LB medium supplemented with 100 µg ml^−1^ ampicillin at 37°C. Heterologous overexpression of ExnV1 was induced with 0.2%(*w*/*v*) l-rhamnose when the culture reached an OD_600 nm_ of 0.3–0.4. Induction was performed for 4 h at 30°C and 200 rev min^−1^. Expression cultures were harvested by centrifugation at 5000*g* for 15 min at 4°C. The collected cell pellets were stored frozen until the purification of the produced recombinant proteins.

### 2.3. Protein purification

Harvested cells containing recombinant PolI_G20c or ExnV1 were lysed by ultrasonication using a UP400s homogenizer (Hielscher Ultrasound Technology). The lysates were separated from cell debris by centrifugation at 14 000*g* for 30 min at 4°C. The supernatants were filtered through regenerated cellulose 0.22 µm pore-size filters (GE Healthcare Life Sciences). His-tagged PolI_G20c and ExnV1 were purified by immobilized metal ion affinity chromatography (IMAC), with nickel as a ligand, using HisTrap columns (GE Healthcare Life Sciences) and an ÄKTA start FPLC purification system (GE Healthcare Life Sciences). Recombinant PolI_G20c bound to the resin in binding buffer [100 m*M* Tris–HCl pH 7.4, 500 m*M* NaCl, 5%(*v*/*v*) glycerol] was eluted with a linear gradient to 100% elution buffer [100 m*M* Tris–HCl pH 7.4, 500 m*M* NaCl, 500 m*M* imidazole, 5%(*v*/*v*) glycerol].

Recombinant ExnV1 bound to the resin in binding buffer [50 m*M* Tris–HCl pH 7.4, 500 m*M* NaCl, 60 m*M* imidazole, 10%(*v*/*v*) glycerol] was eluted with elution buffer [50 m*M* Tris–HCl pH 7.4, 500 m*M* NaCl, 500 m*M* imidazole, 10%(*v*/*v*) glycerol]. Buffer exchange was performed by dialysis. The purity and integrity of PolI_G20c and ExnV1 were assessed by glycine SDS–PAGE using 4–20% gradient gels. The protein concentration was determined considering the theoretically calculated extinction coefficients by measuring the absorbance at 280 nm (*A*_280_) using a NanoDrop 1000 spectrophotometer (Thermo Fisher Scientific).

### 2.4. Screening for polymerase and 3′–5′ exonuclease activity of PolI_G20c

The polymerase activity of PolI_G20c was assessed as described by Schrier & Wilson (1976 ▸) with minor modifications. PolI_G20c was diluted with dilution buffer [10 m*M* Tris–HCl pH 7.5, 50 m*M* KCl, 1%(*v*/*v*) glycerol, 0.1%(*v*/*v*) Triton X-100] to achieve an incorporation rate inside the linear range of the activity assay, resulting in a final concentration of 0.02 µg µl^−1^. The reaction mixtures were incubated at temperatures ranging from 20 to 85°C in reaction buffer [50 m*M* Tris–HCl pH 7.9 for 20–45°C, pH 8.5 for 40–65°C or pH 9.1 for 60–85°C, 50 m*M* KCl, 5 m*M* MgCl_2_, 33 µ*M* of each dNTP, 0.125 µ*M* [^3^H]-dTTP (American Radiolabeled Chemicals)]. The reaction buffer was supplemented with herring sperm DNA (Promega) to compensate for temperature effects on pH and substrate availability up to 0.7 mg ml^−1^ denatured herring sperm DNA for 20–65°C and up to 4 mg ml^−1^ for 60–85°C. Reactions were terminated after 10 min incubation with 10 m*M* EDTA. The reaction mixtures were then precipitated on Whatman glass fibre mats (Cytiva) with 5%(*w*/*v*) trichloroacetic acid. Radioactive decay was assessed using a scintill­ation cocktail and a scintillation counter (PerkinElmer). The temperature profile of PolI_G20c was compared with the thermoactivity of IsoPol BST^+^ DNA polymerase (ArcticZymes Technologies) and of GoTaq DNA polymerase (Promega).

The 3′–5′ exonuclease activity of PolI_G20c was assessed by applying a method based on activity measurements both in the absence and the presence of 4.7 m*M* cold nonradioactive dNTPs with a [^3^H]-dNTP-labelled double-strand PCR fragment serving as a substrate. PolI_G20c was diluted with dilution buffer [10 m*M* Tris–HCl pH 7.5, 150 m*M* NaCl, 1%(*v*/*v*) glycerol, 0.1%(*v*/*v*) Triton X-100]. The reaction mixtures were incubated at 65°C in reaction buffer (20 m*M* Tris–HCl pH 8.5, 50 m*M* NaCl, 5 m*M* MgCl_2_, 4.7 m*M* dNTP or 27 m*M* Tris–HCl pH 8.5, 67 m*M* NaCl, 6.7 m*M* MgCl_2_, 4.7 m*M* dNTP). Phusion High-Fidelity DNA polymerase (New England Biolabs) diluted with dilution buffer was used as a positive control. The exonuclease activity was assessed by measuring the release of soluble nonprecipitating radioactive nucleotides with a MicroBeta^2^ microplate counter (Perkin­Elmer). High radioactivity release in the absence of nucleotides and a lack of radioactivity release in the presence of nucleotides confirms 3′–5′ exonuclease activity, as cold dNTPs effectively inhibit exonuclease activity.

### 2.5. Crystallization and X-ray diffraction data collection of PolI_G20c and ExnV1

The buffer of ExnV1 was exchanged from 50 m*M* Tris–HCl pH 7.4, 500 m*M* NaCl, 500 m*M* imidazole, 10%(*v*/*v*) glycerol to 20 m*M* Tris–HCl pH 7.4, 500 m*M* NaCl, 10%(*v*/*v*) glycerol. Differential scanning fluorimetry showed that ExnV1 was stabilized by thymidine monophosphate (TMP) and MgCl_2_, which was added to the buffer prior to concentration. Crystals appeared in the condition 100 m*M* HEPES–NaOH pH 7.5, 200 m*M* MgCl_2_, 13%(*v*/*v*) PEG Smear Medium, 5%(*v*/*v*) 2-propanol, 5%(*v*/*v*) glycerol and a seed stock was prepared. Co-crystallization with a terbium compound for use in experimental phasing was obtained by adding 100 µl protein solution to 0.6 mg of the terbium cluster compound Crystallophore (Molecular Dimensions; Engilberge *et al.*, 2017 ▸). Crystals were obtained by setting up micro matrix-seeded crystallization experiments (D’Arcy *et al.*, 2007 ▸) using a protein concentration of 13.9 mg ml^−1^ and the BCS screen (Molecular Dimensions). Crystallization experiments were set up as sitting drops in MRC 3-well plates using a Mosquito robot (TTP Labtech) at 20°C. Crystals appeared in the following condition: 100 m*M* Bicine pH 9.3, 30%(*v*/*v*) PEG Smear Low. Crystals were flash-cooled in liquid nitrogen after addition of a cryo-solution consisting of 100 m*M* Bicine pH 9.3, 30%(*v*/*v*) PEG Smear Low, 5 m*M* TMP, 10 m*M* MgCl_2_, 20%(*v*/*v*) PEG 400.

Prior to the crystallization of PolI_G20c, a buffer exchange was performed by changing the buffer from 100 m*M* Tris–HCl pH 7.4, 500 m*M* NaCl, 250 m*M* imidazole, 5%(*v*/*v*) glycerol to 20 m*M* sodium cacodylate pH 6.7, 150 m*M* NaCl. The protein was concentrated to 6.4 mg ml^−1^ and mixed with 5 m*M* TMP and 10 m*M* MgCl_2_. The initial crystals were obtained from a JCSG+ screen condition (100 m*M* Tris–HCl pH 8.5, 1.26 *M* ammonium sulfate, 200 m*M* lithium sulfate). A seed stock was prepared from these crystals and was used for further optimization. The crystals used for data collection appeared in the condition 100 m*M* Tris–HCl pH 8, 1.3 *M* ammonium sulfate, 50 m*M* Li_2_SO_4_, 20 m*M* MgCl_2_; prior to crystallization the reservoir was supplemented with 2 m*M* DTT. The crystals were flash-cooled in liquid nitrogen after addition of a cryo-solution consisting of 100 m*M* Tris–HCl pH 8, 1.3 *M* ammonium sulfate, 50 m*M* lithium sulfate, 25%(*v*/*v*) glycerol, 20 m*M* MgCl_2_, 5 m*M* TMP.

### 2.6. Data collection, structure solution and refinement of ExnV1 and PolI_G20c

For ExnV1, data were collected at 100 K at station I03 of Diamond Light Source, Didcot, UK (λ = 1.6488 Å) equipped with an EIGER2 XE 16M detector. Data were collected with a rotation range of 0.1° per image and an exposure time of 10 ms per image. The anomalous data were processed in *XDS* (Kabsch, 2010 ▸) and scaled in *XSCALE*. *AIMLESS* (Evans & Murshudov, 2013 ▸) was used to produce an MTZ file that was fed into the *CRANK*2 pipeline (Pannu *et al.*, 2011 ▸), which uses *SHELXC*/*D*/*E* (Sheldrick, 2010 ▸) for automatic phasing by single-wavelength anomalous diffraction (SAD). One copy of the protein was found in the asymmetric unit with one terbium cluster bound per molecule. Refinement was performed with *BUSTER* (Bricogne *et al.*, 2011 ▸) using TLS parametrization in the modelling of *B* factors. Model building was carried out in *Coot* (Emsley *et al.*, 2010 ▸). Data-collection and refinement statistics are shown in Table 1 ▸.

For PolI_G20c, data were collected at 100 K at station I03 of Diamond Light Source, Didcot, UK (λ = 0.9762 Å) equipped with an EIGER2 XE 16M detector. Data were collected with a rotation range of 0.1° per image and an exposure time of 4 ms per image. The data set was integrated by the expert system *autoPROC* (Vonrhein *et al.*, 2011 ▸) using *XDS* (Kabsch, 2010 ▸) and utilizing *STARANISO* anisotropic scaling (Tickle *et al.*, 2018 ▸), which gave diffraction limits of 2.9, 3.1 and 3.9 Å in the three principal directions of diffraction.

The structure was determined using the *Phaser* molecular-replacement software (McCoy *et al.*, 2007 ▸). The structure of ExnV1 and PDB entry 4bwm (Blatter *et al.*, 2013 ▸) were used as search models. Two copies of the protein (*A* and *B*) were found in the asymmetric unit. The stretch comprising residues 326–376 in both the *A* and *B* chains was built using *Coot* and the corresponding residues of PDB entries 1kln (Beese *et al.*, 1993 ▸), 1d9d and 1d8y (Teplova *et al.*, 1999 ▸). The structure was refined in *BUSTER* (Bricogne *et al.*, 2011 ▸) using a sequence number-modified model of the ExnV1 external target for geometrical restraints (Smart *et al.*, 2012 ▸) for chains *A* and *B*. In the late stages of model building and refinement five models were generated using the *ColabFold* (Mirdita *et al.*, 2017 ▸, 2019 ▸, 2021 ▸; Mitchell *et al.*, 2020 ▸; Steinegger *et al.*, 2019 ▸) interface to the *AlphaFold*2 (AF2; Jumper *et al.*, 2021 ▸) automatic 3D folding software using the sequence of PolI_G20c as input. The option to use template structures was selected and 518 known 3D structures from the PDB were used in model generation. The AF2 model with the highest predicted mean lDDT (Mariani *et al.*, 2013 ▸; model 1, with a mean lDDT of 93.2) was chosen and compared with the current X-ray model. The initial C^α^ root-mean-square deviation (r.m.s.d.) after *SSM* (Krissinel & Henrick, 2004 ▸) superimposition was 1.70 Å for 667 residues. After local least-squares superimposition of parts of the AF2 model with the X-ray model, two residue stretches from the AF2 model (327–386 and 518–536) were chosen to replace the residues of the X-ray model in both the *A* and *B* chains. Those residues were thereafter subjected to real-space refinement in *Coot* towards the electron density and finally to reciprocal-space refinement in *BUSTER*. Data-collection and refinement statistics are shown in Table 1 ▸.

### 2.7. Bioinformatic tools and softwares

The theoretical pI value and molecular mass of the protein were estimated using the ExPASy server (https://web.expasy.org/compute_pi/; Gasteiger *et al.*, 2005 ▸). Sequence-similarity search, conserved domain identification and initial distance-tree analysis were made in NCBI *BlastP* (https://blast.ncbi.nlm.nih.gov/; NCBI Resource Coordinators, 2016 ▸) against the nonredundant protein sequence database, excluding Models XM/XP and uncultured/environmental sample sequences, using default algorithm parameters. Alignments, pairwise alignment scores and alignment colouring were performed and collected with *Jalview* version 2.11.14 (Waterhouse *et al.*, 2009 ▸), using the *Clustal* web service, default settings and *ClustalX* colouring, unless stated otherwise. Evolutionary analysis was performed and a phylogenetic tree was obtained with *MEGA* version X (Kumar *et al.*, 2018 ▸) applying the maximum-likelihood method and a JTT matrix-based model (Jones *et al.*, 1992 ▸) under default parameter values with 500 bootstrap replications. Sequences were collected from a search in NCBI *BlastP* along with the manually added sequences of PolI_Tth15-6 and ExnV1. Protein attributions to (super)families were performed with InterPro version 83.0 (https://www.ebi.ac.uk/interpro; Blum *et al.*, 2021 ▸). All illustrations of protein structures were prepared with *CCP*4*mg* (McNicholas *et al.*, 2011 ▸) and previous structures were collected from the PDB (Berman *et al.*, 2003 ▸). Conservative sequence motifs were investigated in the Conserved Domain Database (CDD) version 3.18 (Lu *et al.*, 2020 ▸). Protein structure comparisons were made with PolI_G20c chain *A* (PDB entry 7r0k) and ExnV1 (PDB entry 7r0t) in the *DALI* server (https://ekhidna2.biocenter.helsinki.fi/dali; Holm, 2020 ▸) against the full PDB.

## 3. Results and discussion

### 3.1. Production and purification of PolI_G20c and ExnV1

**Figure 1:**
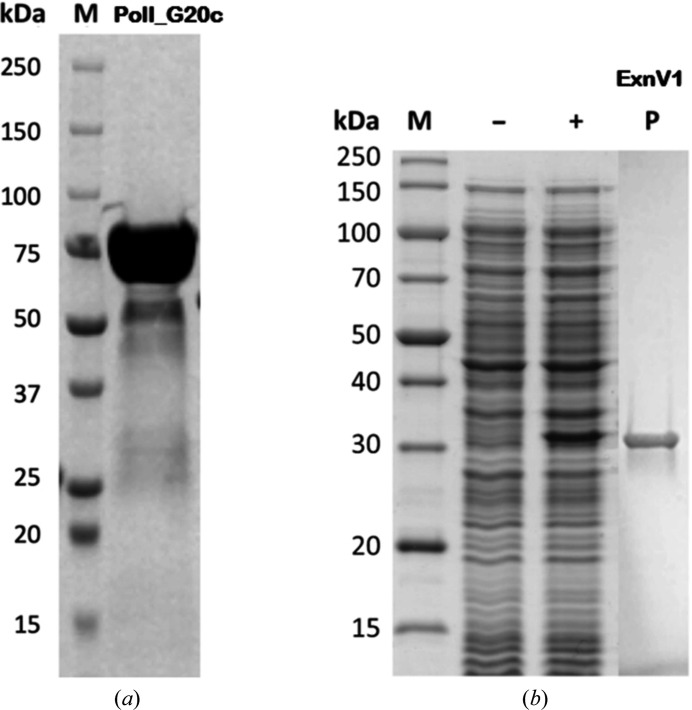
Production and purity of PolI_G20c and ExnV1. (*a*) Molecular-weight and purity assessment of DNA polymerase I from *Thermus* phage G20c (PolI_G20c). Lane M, Precision Plus Protein Dual Colour Standards (Bio-Rad) molecular-mass marker; lane PolI_G20c, recombinant PolI_G20c purified by IMAC. (*b*) Production, purity and molecular-weight assessment of ExnV1. Lane M, PageRuler Unstained Broad Range Protein Ladder (Thermo Fisher Scientific); lane −, non-induced soluble crude extract of the ExnV1 expression culture; lane +, induced soluble crude extract of the ExnV1 expression culture; lane P, recombinant ExnV1 purified by IMAC.

The gene encoding PolI_G20c originates from *Thermus* phage G20c, which was first isolated in Russia (Xu *et al.*, 2017 ▸). In this study, PolI_G20c was successfully produced in *E. coli* Origami (DE3) cells in shake-flask cultivations with a maximum productivity *Q* = 0.36 g l^−1^ h^−1^. The deduced amino-acid sequence of PolI_G20c consists of 728 residues of biological relevance and a C-terminal hexahistidine tag. The calculated molecular weight and pI value, excluding the tag, are 82.4 kDa and 5.85, respectively (inclusion of the tag corresponds to a molecular weight and pI of 83.5 kDa and 5.98, respectively), consistent with the molecular weight detected by SDS–PAGE analysis of pure recombinant PolI_G20c obtained after a single IMAC step (Fig. 1 ▸ *a*).

The gene coding for ExnV1 originates from a metagenome collected within the Virus-X project (Aevarsson *et al.*, 2021 ▸). It has a length of 942 bp (corresponding to 314 residues), and sequence comparison using *BlastP* and the CDD revealed that ExnV1 only contains the putative 3′–5′ exonuclease domain typical of DNA polymerases I, indicating that ExnV1 may be a truncated DNA polymerase I.

A synthetic gene, codon-optimized for expression in *E. coli*, resulted in high yields of recombinant protein in *E. coli* strain JM109. The maximum productivity of ExnV1 obtained in shake-flask cultivation was *Q* = 0.65 g l^−1^ h^−1^. ExnV1 showed no oligomerization and was soluble both in the cytoplasm of the expression strain and in the crude extract after cell lysis. SDS–PAGE demonstrated the expected molecular weight of about 36 kDa (Fig. 1 ▸ *b*). ExnV1 remained soluble after purification by IMAC and did not aggregate, resulting in successful purification to near-homogeneity (Fig. 1 ▸ *b*).

### 3.2. Polymerase and 3′–5′ exonuclease activity of PolI_G20c

**Figure 2:**
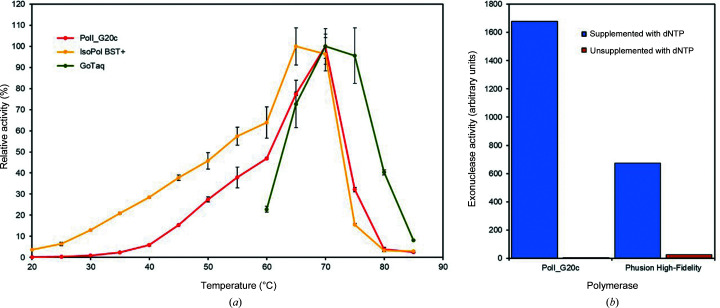
Determination of the activity of PolI_G20c. (*a*) Detection of polymerase activity of PolI_G20c at temperatures ranging from 20 to 85°C and comparison with the activity of IsoPol BST^+^ and GoTaq polymerase. (*b*) Detection of 3′–5′ exonuclease activity of PolI_G20c and comparison with the activity of Phusion High-Fidelity polymerase exonuclease.

The temperature range for polymerase activity of PolI_G20c was assessed as described in Section 2 and was compared with those of the reference polymerases IsoPol BST^+^ and GoTaq (Fig. 2 ▸ *a*). The maximum activity of PolI_G20c and GoTaq was measured at about 70°C, while IsoPol BST^+^ exhibits maximum activity at 65°C. IsoPol BST^+^ also exhibited significant activity at temperatures below 40°C, whereas PolI_G20c displayed very limited activity below this temperature. Hence, PolI_G20c exhibited a significantly narrower temperature range compared with the commercial polymerase.

IsoPol BST^+^ lacks both 5′–3 and 3′–5′ exonuclease activity, while GoTaq Polymerase is a polymerase that lacks 3′–5′ exonuclease activity. To prove the presence of 3′–5′ exo­nuclease activity of PolI_G20c (as predicted from sequence analysis; see below), another reference polymerase (Phusion High-Fidelity) was included for comparison. In the analysis, the fact that the 3′–5′ exonuclease activity of DNA polymerases can be turned off by adding nucleotides to the reaction mixture was used, as previously demonstrated for T4 DNA polymerase (Hershfield & Nossal, 1972 ▸). The presence of nucleotides in the reaction mixture initiates the polymerase activity of such polymerases, which ‘overrides’ the exo­nuclease activity that acts in the opposite direction. In the present study, this effect could also be detected in PolI_G20c when compared with the temperature-stable Phusion High-Fidelity polymerase. Hence, it was concluded that 3′–5′ exonuclease activity was present in PolI_G20c (Fig. 2 ▸ *b*).

### 3.3. Phylogenetic and domain analysis of PolI_G20c and ExnV1

**Figure 3:**
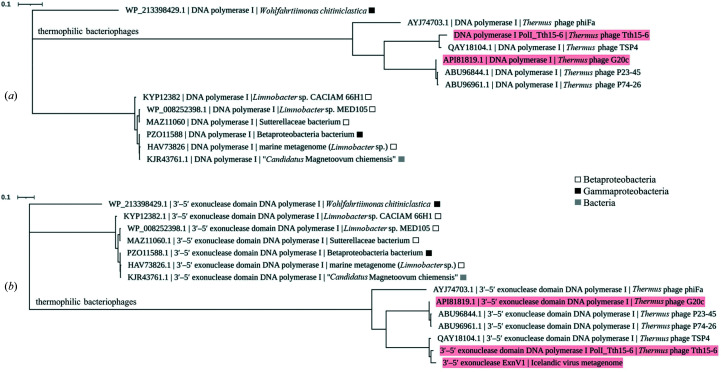
Evolutionary trees displaying the relationships (*a*) of PolI_G20c to PolI_Tth15-6 and deposited sequences encoding DNA polymerases I from thermophilic bacteriophages and (*b*) of ExnV1 to the 3′–5′ exonuclease domain in the closest related DNA polymerase I sequences. The trees are presented to scale, with branch lengths measured in number of substitutions per site. The trees were prepared with *MEGA* version X and were graphically edited with *iTOL* and *Microsoft PowerPoint*.

Sequence analysis of PolI_G20c revealed that the enzyme has a sequence identity of 61% or greater to ExnV1 and to putative DNA polymerases I encoded in the genomes of a group of related phages that have been isolated from different parts of the world (Jasilionis *et al.*, in preparation). The related phages are *Thermus* virus P23-45 (GenBank accession ABU96844.1), *Thermus* virus P74-26 (GenBank accession ABU96961.1; Minakhin *et al.*, 2008 ▸), *Thermus* phage TSP4 (GenBank accession QAY18104.1; Lin *et al.*, 2010 ▸), *Thermus* phage phiFa (GenBank accession AYJ74703.1) and *Thermus* phage Tth15-6. The similarity to other deposited sequences annotated as polymerases was significant lower, with a sequence identity of 31% or below. Evolutionary trees were constructed based on the relationship of PolI_G20c to the closest related protein and PolI_Tth15-6 (Fig. 3 ▸ *a*) as well as the relationship of ExnV1 to 3′–5′ exonuclease domain sequences from the most closely related DNA polymerases I (Fig. 3 ▸ *b*). An alignment of the putative proteins (Supplementary Fig. S1) also confirmed the expected close relationship of PolI_G20c and PolI_Tth15-6 to the putative DNA polymerases I from thermophilic bacteriophages, corroborating the suggestion that ExnV1 is likely to originate from a related thermophilic bacteriophage.

**Figure 4:**
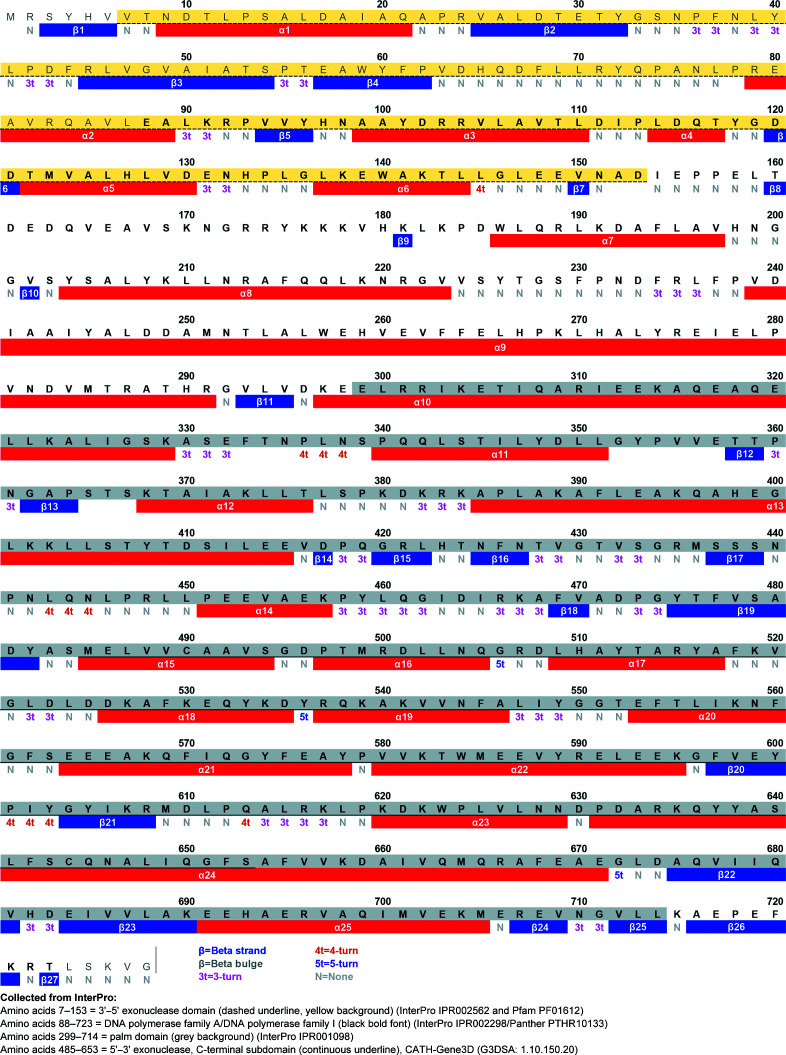
Domains found in InterPro for the amino-acid sequence and secondary structure of PolI_G20c as seen in *CCP*4*mg* for chains *A* and *B* in PDB entry 7r0k. N indicates residues that are not shown in either chain *A* or *B*.

Sequence-based domain analysis was subsequently made using the deduced amino-acid sequences of PolI_G20c and ExnV1. According to InterPro, the sequence corresponding to amino-acid residues 88–723 of PolI_G20c belongs to DNA polymerase family A (InterPro accession IPR002298) and DNA polymerases I (Panther accession PTHR10133) (marked in bold in Fig. 4 ▸). Within this sequence, residues 299–714 (grey background in Fig. 4 ▸) correspond to the palm domain (InterPro accession IPR001098) and residues 7–153 (underlined with a dotted line) correspond to the 3′–5′ exonuclease domain, where approximately half of the domain is included in the sequence identified in the DNA polymerase A family (Fig. 4 ▸). The 3′–5′ exonuclease domain, including the residues stretching forward to the start of the palm domain (residues 4–284), is also attributed to belong to the homologous ribonuclease H-like superfamily. This area is also present in ExnV1, corresponding to residues 3–287 of a total of 314 amino acids. This domain is, for example, also present in DNA polymerase I from *E. coli*, but is missing in DNA polymerase I from *T. aquaticus* (*Taq* polymerase; UniProt accession P19821 and PDB entry 1bgx; Kim *et al.*, 1995 ▸), which on the other hand encodes the typical 5′–3′ exonuclease domain. The 5′–3′ exonuclease domain includes a first part (or subdomain) at the N-terminus and a second part at the C-terminus. Interestingly, the N-terminal subdomain of the 5′–3′ exonuclease domain is not present in PolI_G20c, while the C-terminal subdomain comprising residues 485–653 (underlined in Fig. 4 ▸) can be found based on data collected from the unintegrated Cathedral database, where it is considered to be involved in interactions with DNA and proteins (InterPro accession IPR036279).

### 3.4. Overall crystal structures of ExnV1 and PolI_G20c

**Figure 5:**
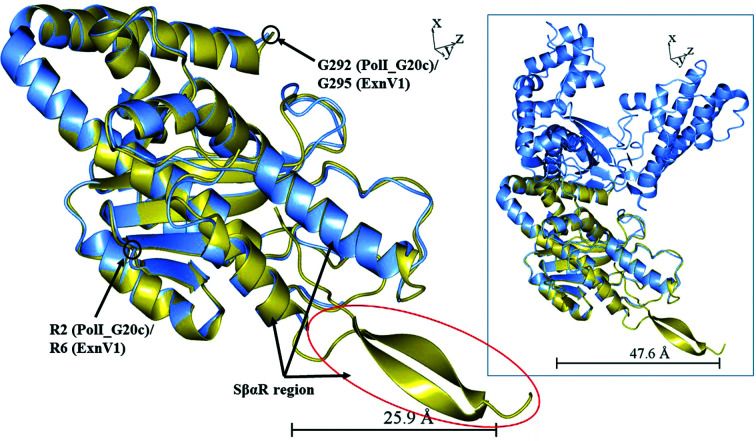
ExnV1 (gold) is superposed on chain *B* of PolI_G20c (ice blue, fixed model). The image in the box displays the full length of PolI_G20c chain *B*, whereas the close-up image displays residues 2–292 of PolI_G20c chain *B* and residues 6–295 of ExnV1. The two β-strands that are only seen in ExnV1 are part of the novel specific structure motif SβαR and are encircled in red. The images were prepared in *CCP*4*mg*.

The crystal structure of ExnV1 contained one polypeptide chain and the overall structure was determined to 2.19 Å resolution using the co-crystallized terbium compound. Attempts to crystallize seleno-l-methionine-derivatized PolI_G20c (or crystallization with terbium as above) were not successful, but the structure of PolI_G20c could be determined using the ExnV1 structure combined with PDB entry 4bwm (Blatter *et al.*, 2013 ▸), as described in Section 2. The structure of PolI_G20c consists of two polypeptide chains, *A* and *B*, that could possibly be arranged as a dimer according to *PISA* structure analysis in *CCP*4*mg*. ExnV1 corresponds to the first 315 residues of PolI_G20c (Supplementary Fig. S1), and superposition of ExnV1 on PolI_G20c (with an r.m.s.d. of 1.01 Å over 236 residues) shows that the structures of the two proteins match each other very well (Fig. 5 ▸). Residues 152–185 are missing in chain *B* of PolI_G20c; however, the residues corresponding to those in ExnV1 are present and from the sequence alignment it is interpreted that PolI_G20c also resembles ExnV1 in this area, which is part of a novel structure motif (SβαR) that is discussed further below. The major difference between the two proteins is that ExnV1 only comprises the smaller 3′–5′ exonuclease, whereas PolI_G20c also includes the larger palm domain that is responsible for the polymerase activity.

### 3.5. Structure of PolI_G20c

**Figure 6:**
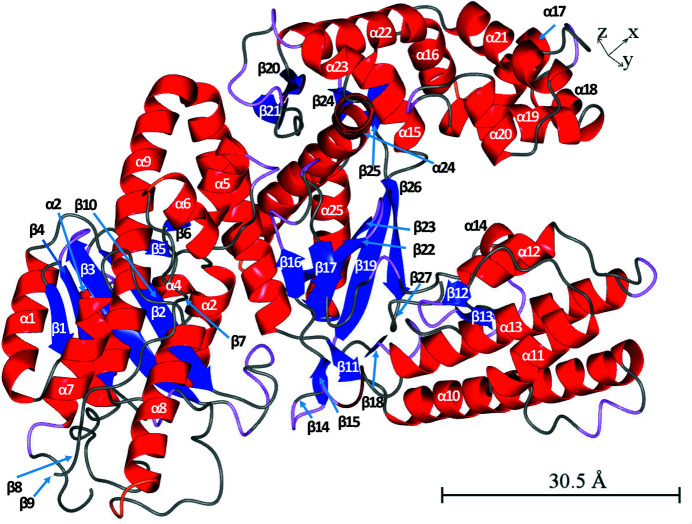
Secondary structure of the DNA polymerase I PolI_G20c. The image was prepared in *CCP*4*mg* with PolI_G20c chain *A* (PDB entry 7r0k).

The secondary-structure elements in PolI_G20c are displayed in Fig. 6 ▸. It contains 25 α-helices and 27 β-strands as identified in *CCP*4*mg* based on both chains *A* and *B* in PDB entry 7r0k for PolI_G20c.

**Figure 7:**
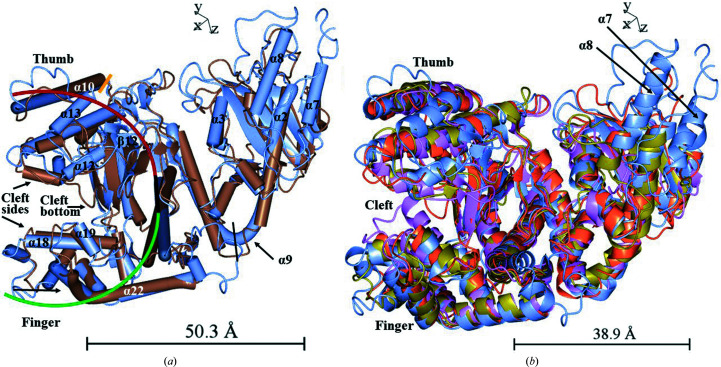
(*a*) The Klenow fragment (KF; PDB entry 1d9f; chain *A* in brown) is superposed on PolI_G20c (PDB entry 7r0k; chain *A* in ice blue) displayed as tubes. The black line indicates the border between the small and large domain as described by Ollis *et al.* (1985 ▸) and the orange line indicates where the larger, palm domain starts at residue 299 in PolI_G20c and residue 228 in KF (corresponding to residue 551 in full-length DNA polymerase I from *E. coli*) according to InterPro. (*b*) Superposition of the best-matching structures based on a search using the *DALI* server. Chain *A* of DNA polymerase I from *G. stearothermophilus* (BF; PDB entry 1knc, gold), KF (PDB entry 2kzz, orange) and *Taq* polymerase (PDB entry 5ytf, violet) are superposed on chain *A* of PolI_G20c (ice blue) as ribbons. The structures match very well; however, α-helices 7 and 8 in PolI_G20c differ from all of the other compared enzymes and are part of a novel motif. The images were prepared with *CCP*4*mg*.

Analysis of structural similarity using the *DALI* server (https://ekhidna2.biocenter.helsinki.fi/; Holm, 2020 ▸; Dawson *et al.*, 2017 ▸) against the PDB generated 65 unique PDB entries that had a *Z*-value of between 30 and 33.3. The best matches were two structures of DNA polymerase I from the thermophilic bacterium *G. stearothermophilus* (BF) bound to a DNA substrate (PDB entries 1nkc and 6mu4; UniProt accession P52026; Jackson *et al.*, 2019 ▸; Johnson & Beese, 2004 ▸), the Klenow fragment from *E. coli* DNA polymerase I (PDB entry 2kzz; UniProt accession P00582; Brautigam *et al.*, 1999 ▸) and the thermostable *Taq* polymerase (PDB entry 5ytf; UniProt accession P19821; Zeng *et al.*, 2019 ▸). In Fig. 7 ▸(*a*) the Klenow fragment (PDB entry 1d9f, chain *A*; brown; Teplova *et al.*, 1999 ▸) is superposed on PolI_G20c chain *A* (ice blue, fixed model), while in Fig. 7 ▸(*b*) the *G. stearothermophilus* polymerase, the Klenow fragment and *Taq* polymerase are all superposed on PolI_G20c.

It is known that the mechanism of DNA polymerase I includes a substrate-dependent conformational shift involving a substrate-coordinating tyrosine residue in the O-helices area (Li *et al.*, 1998 ▸), corresponding to Tyr549 and α-helices 18, 19, 20 and 21 in PolI_G20c (see also Section 3.5.1). Despite this, all of the best-matching deposited structures were of DNA polymerase I crystallized with DNA (Fig. 7 ▸ *b*), even though the 3D structure of PolI_G20c (PDB entry 7r0k) was not. The structural resemblance is striking, with the exception of α-helices 7 and 8 of PolI_G20c, which differ from the other compared enzymes and are part of the novel motif (SβαR; further described in Section 3.5.2). These helices are situated in the more nonconserved part of the enzymes between the 3′–5′ exo­nuclease domain and the palm domain, as displayed in Fig. 7 ▸, or possibly within the 3′–5′ exonuclease domain, as discussed below.

The Klenow fragment is a well described enzyme that is known to have one small domain (approximately the first 200 residues of a total of 605 residues) that mostly comprises parallel β-pleated strands with α-helices on both sides and one larger domain that resembles a right hand with fingers and a thumb (Ollis *et al.*, 1985 ▸). The overall structures of PolI_G20c and the Klenow fragment were similar, with the same structural orientation of the smaller domain that contains the active-site residues of the 3′–5′ exonuclease domain (Derbyshire *et al.*, 1988 ▸; discussed further in Section 3.5.2). The larger C-terminal palm domain (indicated with a red, black and green curve) contains the active-site residues of the polymerase domain (Fig. 7 ▸ *a*). Also, the orientation of the polymerase subdomains, for example the finger (at the red end of the curve) and thumb (at the green end of the curve) and the deep cleft with β-strands in its bottom between the sub­domains, fits very well. A difference is seen in α-helix 9 of PolI_G20c, which bends in a V-like shape, rather than being divided into two separate helices (as in the Klenow fragment). However, one could interpret α-helix 9 of PolI_G20c as two separate helices (9a and 9b) since the residue (Pro268) at the bottom of the bend is a proline, which could act as a helix breaker.

#### 3.5.1. Palm domain of PolI_G20c

**Figure 8:**
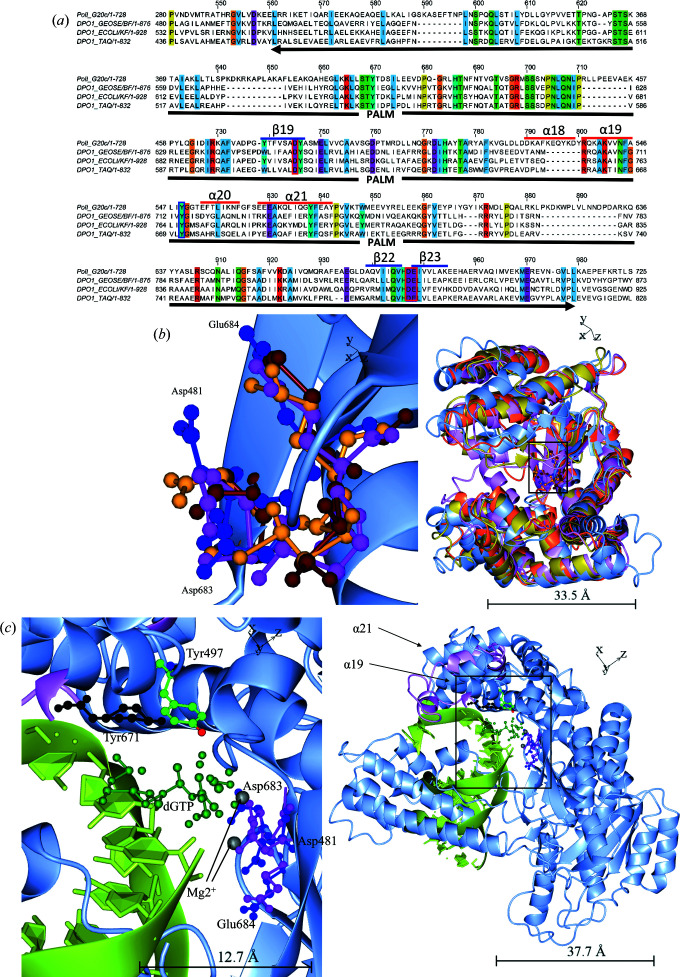
Palm domain with active-site residues of PolI_G20c compared with the Klenow fragment, DNA polymerase I from *G. stearothermophilus* and *Taq* polymerase. (*a*) Alignment of PolI_G20c with DNA polymerase I from *G. stearothermophilus*, *E. coli* DNA polymerase I and *Taq* polymerase generated in *Jalview*. Conserved residues (threshold 100%) are coloured according to *ClustalX*. The catalytic triad residues are marked with red boxes. α-Helices 18, 19, 20 and 21 of PolI_G20c, situated between the catalytic residue Asp481 and the two catalytic residues Asp683 and Glu684 in PolI_G20c, are predicted to make the same type of conformational shift, often described in the literature, as made by O-helices in the homologous polymerases. The substrate-coordinating residue Tyr549 is marked with a blue box and the whole palm domain that includes the finger and thumb subdomains as defined in InterPro is underlined with a black arrow. (*b*) Residues from the palm domain, chain *A* of BF (PDB entry 1knc, gold), the Klenow fragment (PDB entry 2kzz, orange) and *Taq* polymerase (PDB entry 5ytf, violet) are superposed and displayed on the palm domain of PolI_G20c chain *A* (ice blue). The catalytic amino-acid triad at the bottom of the cleft is displayed in ball-and-stick representation and is also enlarged, where Asp683, Glu684 and Asp481 in PolI_G20c are blue and the corresponding residues are violet in *Taq* polymerase, brown in DNA polymerase I from *E. coli* and dark gold in BF. (*c*) The catalytic residues of *Taq* polymerase (violet) are superimposed on the corresponding residues of PolI_G20c and the two Mg^2+^ ions from the *Taq* model (PDB entry 5ytf) are displayed in grey, along with the DNA substrate in light green, dGTP in dark green and Tyr671 in black, corresponding to Tyr549 in PolI_G20c, which is coloured according to standard atom colours. Part of the finger domain of *Taq* polymerase is also displayed in violet. The images were prepared with *CCP*4*mg*.

The palm domain of PolI_G20c was compared with the top hits from the *DALI* server. The alignment of the palm domain (conserved residues coloured according to *ClustalX*, threshold 100%; Fig. 8 ▸ *a*) indicates that all of the enzymes should share the same mechanism. To pinpoint similarities and differences in the structural conservation of catalytic residues, only the palm domains, as defined in InterPro for each enzyme, are superposed on PolI_G20c in Fig. 8 ▸(*b*). The catalytic amino-acid triad for polymerase activity (Li *et al.*, 1998 ▸) at the area of the bottom of the cleft is displayed and enlarged in Fig. 8 ▸(*b*) and the catalytic residues correspond to Asp683, Glu684 and Asp481 in PolI_G20c (marked with red boxes in Fig. 8 ▸ *a*). It is clearly seen that the orientation of the catalytic triad of PolI_G20c resembles that in the other polymerases, which strengthens the argument for a similar polymerase mechanism. This mechanism involves one Mg^2+^ ion that promotes deprotonation of the 3′-OH of the primer strand and assists the leaving of the pyrophosphate, and another Mg^2+^ ion that stabilizes the formation of a pentacovalent transition state at the α-phosphate by facilitating the formation of a 90° O—P—O bond angle; this ion also facilitates the leaving of pyrophosphate (Steitz, 1993 ▸).

In Fig. 8 ▸(*c*) the catalytic residues of *Taq* polymerase are superimposed on the corresponding residues of PolI_G20c and the two Mg^2+^ ions from the *Taq* polymerase model (PDB entry 5ytf), along with DNA and a dGTP. The image also displays Tyr671 in *Taq* polymerase, which corresponds to Tyr549 in PolI_G20c. The tyrosine residues from the respective enzymes are situated a little apart from each other, which is not unexpected since the conformation of the finger domain differs in *Taq* polymerase (partly displayed in violet) compared with PolI_G20c, which was crystallized without substrate. Several more residues are involved in the binding and coordination of the incoming dNTPs and DNA that are not described here; however, a detailed description of the polymerase mechanism with each step in chronological order has recently been published (Chim *et al.*, 2021 ▸).

#### 3.5.2. 3′–5′ exonuclease domain of ExnV1 and PolI_G20c

**Figure 9:**
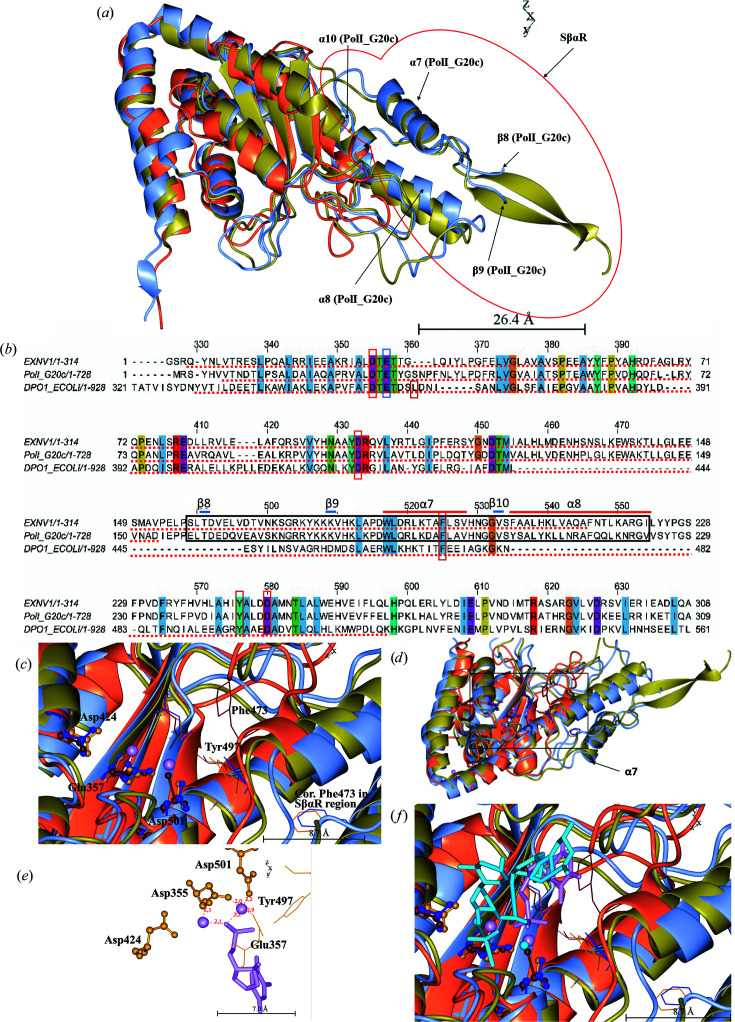
Comparison of the 3′–5′ exonuclease domain of ExnV1, PolI_G20c and the Klenow fragment.(*a*) Residues 339–548 of the Klenow fragment (PDB entry 2kzz, orange) are superposed on the corresponding residues of PolI_G20c (PDB entry 7r0k, ice blue) and ExnV1 (PDB entry 7r0t, gold). The SβαR region of ExnV1 and PolI_G20c is marked with a red ring and includes α-helices 7 and 8 and β-strands 8, 9 and 10 of PolI_G20c, which are marked with arrows. (*b*) Sequence alignment of ExnV1, PolI_G20c and DNA polymerase I from *E. coli* generated in *Jalview* with conserved residues (threshold 100%) coloured according to *ClustalX*. The 3′–5′ exonuclease domain as defined in InterPro is underlined with a red dotted line and the SβαR structural motif is marked with a black box, including α-helices and β-strands of PolI_G20c, which are marked with red and blue lines, respectively, above the alignment. Crucial catalytic residues are marked with red boxes, important residues with secondary roles are marked with orange boxes and Glu357 is marked with a blue box. (*c*) As (*a*) but from another angle and with a box indicating an enlarged area containing crucial catalytic residues shown in ball-and-stick representation and important residues with secondary roles, including Glu357, displayed with fat bonds, where residues of the Klenow fragment, PolI_G20c and ExnV1 are coloured brown, blue and dark gold, respectively. (*d*) An enlarged area of (*c*). (*e*) The direct contact between the metal ions and catalytic residues of ExnV1 (numbered according to the corresponding residues of the Klenow fragment). (*f*) As (*c*) but with the metal ions and substrate from the model of the Klenow fragment displayed in light blue and the nucleotide and metal ions from ExnV1 displayed in violet.

ExnV1 was also investigated using the *DALI* server against the PDB. The search generated 13 matches with a *Z*-value above 20 and all of these models are Klenow fragments of DNA polymerase I from *E. coli*. The best match was with the previously discussed PDB entry 2kzz, with a *Z*-value of 22.9. In Fig. 9 ▸(*a*) residues 339–548 of the Klenow fragment in PDB entry 2kzz (orange) are superposed on the corresponding residues of PolI_G20c (PDB entry 7r0k, ice blue) and ExnV1 (PDB entry 7r0t, gold).

The major difference between the Klenow fragment, PolI_G20c and ExnV1 is that the latter two have a separate structure towards the end of the 3′–5′ exonuclease domain, corresponding to residues 157–222 in ExnV1 and 158–223 in PolI_G20c. This specific structural motif, termed SβαR, consists of two rather long β-strands in ExnV1, followed by two α-helices corresponding to α-helices 7 and 8, with a very short β-strand 10 between them. In PolI_G20c the supposed two long β-strands are only seen as one-residue β-strands 8 and 9, since there is a gap in the structure, although if all of the amino acids in the structure were seen we propose that they would resemble the long corresponding β-strands in ExnV1. The sequence corresponding to the SβαR region from ExnV1 has greater than 65% sequence identity to the SβαR region in PolI_G20c. The separate ExnV1 SβαR sequence was also analysed by *BlastP*, showing that hits were only found in the previously discussed (Section 3.3) homologous putative DNA polymerases I from the related thermophilic bacterio­phages G20c, TSP4, P74-26, P23-45 and PhiFA along with one hypothetical protein from *E. coli*. To our knowledge, no specific structure motif resembling SβαR has previously been connected to DNA polymerase I enzymes. Interestingly, there is a report of a specific exonuclease N-terminal region, NTR (Milton *et al.*, 2016 ▸), from the single DNA polymerase encoded in the apicoplast (apPOL) of the malaria-causing parasite *Plasmodium falciparum*. However, even if the NTR involves two rather large β-strands (PDB entry 5dkt) it cannot be fitted either onto the structure or the amino-acid sequence of ExnV1 or PolI_G20c. The sequence of the SβαR structural motif is displayed in the black box in the alignment in Fig. 9 ▸(*b*), with α-helices and β-strands displayed above the box using the numbering of PolI_G20c as displayed in Fig. 4 ▸. The sequence encoding the SβαR motif appears to interfere with the algorithms predicting the end of the 3′–5′ exonuclease domain in InterPro, compared with the alignment of the sequences in *Jalview* (Fig. 9 ▸ *b*). InterPro defines the SβαR region to be almost completely within the 3′–5′ exonuclease domain in ExnV1, whereas a little surprisingly it is not included in this domain at all in PolI_G20c. From the alignment, it could be interpreted that the Klenow fragment has part of the SβαR region in its 3′–5′ exonuclease domain; however, this is not seen in the structure. Furthermore, some of the proposed crucial catalytic residues, as discussed later (marked with red boxes in Fig. 9 ▸ *b*), are situated outside the 3′–5′ exonuclease domain area as defined by InterPro both in ExnV1 and PolI_G20c. Currently, we do not know whether the SβαR structural motif has any effect on the efficiency of either the polymerase or the 3′–5′ exonuclease activity. PolI_G20c displays both activities, and while the SβαR structure does not seem to affect the polymerase domain structure in the prox­imity of the active site, some alterations were found among residues proposed to be important in the 3′–5′ exonuclease domain (Derbyshire *et al.*, 1991 ▸).

In the Klenow fragment, the mechanism of the 3′–5′ exonuclease activity has been shown to involve two divalent metal ions that may be either Mg^2+^, Mn^2+^ or Zn^2+^ (Freemont *et al.*, 1988 ▸) and play a major role in both substrate binding and catalysis of the exonuclease reaction (Derbyshire *et al.*, 1988 ▸). Mutagenesis studies have shown that Asp355, Asp424 and Asp501 in the Klenow fragment anchor the metal ions (red boxes in Fig. 9 ▸ *b*) and are crucial for catalytic activity. Furthermore, Leu361, Phe473 and Tyr497 (orange boxes in Fig. 9 ▸ *b*) are proposed to have a secondary role in the positioning of the substrate and may also facilitate the melting of a duplex DNA substrate by interacting with the 3′-end in a mechanism in which a nucleophilic attack on the terminal phosphodiester bond is initiated by a hydroxide ion coordinated to one of the enzyme-bound metal ions (Derbyshire *et al.*, 1991 ▸). Glu357 in the Klenow fragment (marked with a blue box) has also been suggested to be a critical catalytic residue (Freemont *et al.*, 1988 ▸; Beese & Steitz, 1991 ▸), although its role is not quite clear (Derbyshire *et al.*, 1991 ▸). In the sequence alignment with PolI_G20c and ExnV1, residues corresponding to Leu361 in the Klenow fragment are not conserved; otherwise, all other proposed residues are situated among the conserved residues (Fig. 9 ▸ *b*). In the structural alignment Asp355, Asp424 and Asp501 of the Klenow fragment (Fig. 9 ▸ *c*, brown ball-and-stick representation) correspond well to the residues of PolI_G20c (blue) and ExnV1 (dark gold). Furthermore, the residues corresponding to Tyr497 and Glu357 of the Klenow fragment (presented in the same colours but as fat bonds) are oriented in almost exactly the same way in all three proteins. However, the residues of PolI_G20c and ExnV1 that seem to correspond to Phe473 in the Klenow fragment in the sequence alignment are situated rather far away on α-helix 7, which is part of the SβαR region in PolI_G20c and ExnV1 (Fig. 9 ▸ *d*).

In Fig. 9 ▸(*e*) the direct contacts between the metal ions and the more important residues as defined by Derbyshire *et al.* (1991 ▸) are displayed. In the current conformation of ExnV1 (PDB entry 7r0t) the associated metals are in direct contact with the residues corresponding to Asp355, Asp501 and Glu357 in the Klenow fragment and the second O atom of the phosphate group of the substrate nucleotide. All distances in the contacts are between 2.0 and 2.3 Å as interpreted in *CCP*4*mg*. Fig. 9 ▸(*f*) is the same as Fig. 9 ▸(*c*), although here the metal ions and substrate are displayed both for the Klenow fragment and ExnV1.

## 4. Concluding remarks

Even though it was not possible to crystallize seleno-l-methionine-derivatized PolI_G20c, native PolI_G20c was successfully produced in *E. coli* Origami (DE3) cells and it was possible to crystallize the product. To enable refinement of the PolI_G20c structure, there was a need to find additional models to complement those already available in the PDB. Hence, ExnV1 was identified, produced and crystallized and its structure was determined. The gene encoding ExnV1 originates from a thermophilic virus metagenome and was found to be phylogenetically closely related to PolI_G20c and homologous putative DNA polymerases encoded in the genomes of related bacteriophages. Since ExnV1 only includes a 3′–5′ exonuclease domain, the gene encoding ExnV1 is most likely to be truncated. It is concluded that PolI_G20c belongs to the DNA polymerase I family but lacks the typical 5′–3′ exonuclease domain. The structure of ExnV1 revealed that the protein has a structural motif, termed SβαR, which we propose to also be present in PolI_G20c. Moreover, based on sequence conservation, the SβαR structural motif is proposed to be present in related phage polymerases encoded in the genomes of the thermophilic bacteriophages TSP4, P74-26, P23-45, Tth15-6 and phiFA. The SβαR structural motif consists of two β-strands followed by two α-helices that are connected to each other via a short β-strand. SβαR is situated towards the end of the 3′–5′ exonuclease domain and seems to affect the orientation of the conserved residue (corresponding to Phe473 in the Klenow fragment) believed to play a role in substrate binding (Derbyshire *et al.*, 1991 ▸). To our knowledge, the SβαR structural motif has not been reported to be associated with any DNA polymerase I elsewhere. However, a different specific exonuclease N-terminal region is found in the DNA polymerase from the apicoplast of the malaria-causing parasite *P. falciparum* (Milton *et al.*, 2016 ▸), indicating the possibility of a common role.

## 5. Data deposition

Atomic coordinates and structure factors for the reported crystal structure of PolI_G20c have been deposited with PDB entry 7r0k and those for the reported crystal structure of ExnV1 have been deposited with PDB entry 7r0t. Raw data have been deposited at https://proteindiffraction.org. The gene for ExnV1 has been deposited with GenBank accession OK094307 and the gene for PolI_Tth15-6 has been deposited with GenBank accession OK037108. The synthetic gene sequence for PolI_G20c has been deposited with GenBank accession ON338039 and the synthetic gene sequence for ExnV1 has been deposited with GenBank accession ON489251.

## Supplementary Material

PDB reference: PolI_G20c, 7r0k

PDB reference: ExnV1, 7r0t

Supplementary Figure. DOI: 10.1107/S2059798322009895/cb5134sup1.pdf

## Figures and Tables

**Table 1:** X-ray diffraction data-collection, structure-determination and refinement statistics for PolI_G20c and ExnV1 Values in parentheses are for the highest resolution shell. Other relevant quality indicators can easily be extracted from the PDB file header.

	PolI_G20c	ExnV1
Resolution (Å)	76.7–2.97 (3.25–2.97)	45.8–2.19 (2.26–2.19)
Anisotropic limits (Å)	3.9, 2.89, 3.1	
Wavelength (Å)	0.97623	1.6488
Space group	*C*2	*P*2_1_22_1_
*a*, *b*, *c* (Å)	309.8, 98.0, 77.6	42.3, 54.1, 171.7
α, β, γ (°)	90, 98.9, 90	90, 90, 90
Spherical completeness (%)	68.9 (14.6)	98.1 (92.0)
Ellipsoidal completeness (%)	92.5 (53.9)	
Multiplicity	7.1 (7.3)	12.7 (12.3)
No. of observations	230652 (32680)	260608 (20566)
No. of unique reflections	11889 (1634)	20363 (1650)
〈*I*/σ(*I*)〉	12.3 (1.5)	12.5 (1.1)
CC_1/2_ (%)	99.9 (55.5)	99.9 (79.2)
*R*_meas_(*I*) (%)	10.3 (148.5)	14.1 (332.5)
Resolution used for refinement (Å)	153.0–2.97 (3.14–2.97)	45.78–2.19 (2.21–2.19)
*R*_model_(*F*) (%)	21.6 (28.9)	23.7 (43.3)
*R*_free_(*F*) (%)	26.1 (33.9)	27.8 (53.1)
No. of non-H atoms	11067	2415
No. of water molecules	0	46
Mean *B* factor, chain *A*, *B* (Å^2^)	110.0, 138.3	62.4
Mean *B* factor, water (Å^2^)	—	55.9
R.m.s.d. from ideal geometry		
Bond lengths (Å)	0.009	0.008
Bond angles (°)	1.0	0.9
Ramachandran plot quality†		
Favoured regions (%)	93.0	97.5
Allowed regions (%)	6.2	2.15
Outliers (%)	0.8	0.35

† Calculated using a local *MolProbity* server.
