# Supplementary material for: Crystal structure of DNA polymerase I from Thermus phage G20c
Source: Acta Crystallogr D Struct Biol. 2022 Oct 27;78(Pt 11):1384–98. doi: 10.1107/S2059798322009895 (PMC9629493; doi:10.1107/S2059798322009895)
Supplement: Supplementary file 1 [file d-78-01384-sup1.pdf]

STRUCTURAL
BIOLOGY

Volume 78 (2022)

Supporting information for article:

Crystal structure of DNA polymerase I from *Thermus* phage G20c

Josefin Ahlqvist, Javier A. Linares-Pastén, Andrius Jasilionis, Martin Welin, Maria Håkansson, L. Anders Svensson, Lei Wang, Hildegard Watzlawick, Arnþór Ævarsson, Ólafur H. Friðjónsson, Guðmundur Ó. Hreggviðsson, Bernd Ketelsen Striberny, Eirin Glomsaker, Olav Lanes, Salam Al-Karadaghi and Eva Nordberg Karlsson

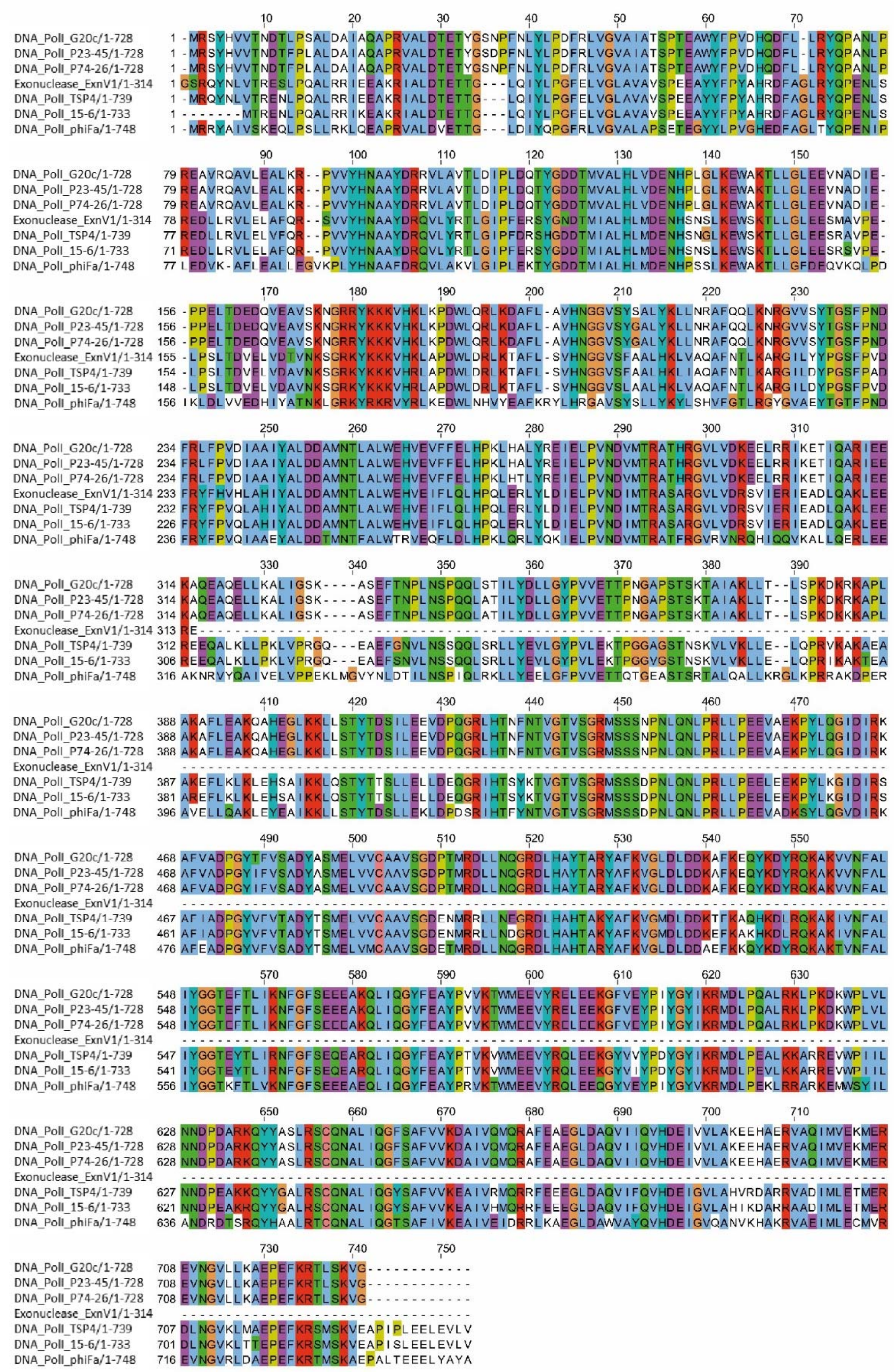

Figure S1 Sequence alignment (in Jalview 2.11.1.4 using Clustal webservice, default setting) between PolI_G20c (728 a.a.), ExnV1 and DNA polymerase I from *Thermus* virus P23-45, *Thermus* virus P74-26, *Thermus* phage TSP4, *Thermus* phage Tth15-6 and *Thermus* phage phiFa.
